# Risk factors of postoperative delirium in the knee and hip replacement patients: a systematic review and meta-analysis

**DOI:** 10.1186/s13018-020-02127-1

**Published:** 2021-01-22

**Authors:** Xiao Rong, Zi-chuan Ding, Hao-da Yu, Shun-Yu Yao, Zong-Ke Zhou

**Affiliations:** Department of Orthopedics, West China Hospital/West China School of Medicine, Sichuan University, 37# Wuhou Guoxue road, Chengdu, 610041 People’s Republic of China

**Keywords:** Knee replacement, Hip replacement, Postoperative delirium, Risk factors

## Abstract

**Background:**

The risk factors of postoperative delirium (POD), a serious while preventable complication, developed by patients undergoing knee and replacement surgery are still under investigation. In this systematic review and meta-analysis, we identified risk factors associated with POD in knee and hip replacement.

**Methods:**

PubMed, Ovid MEDLINE, and Ovid EMBASE were used to identify original researches. The studies evaluating the risk factors of POD after knee and hip replacement were reviewed, and the qualities of the included studies were assessed with Newcastle–Ottawa Scale. Data were extracted, pooled, and a meta-analysis was completed

**Result:**

Twenty-two studies were finally included with a total of 11934 patients who underwent knee or hip replacement and 1841 developed POD with an incidence of 17.6% (95% confidential interval (CI) 13.2–22.0%). Eighteen significant risk factors were identified including advanced age (odds ratio (OR) 1.15 95% CI 1.08–1.22), cognitive impairment (OR 6.84, 95% CI 3.27–14.33), history of cerebrovascular events (OR 2.51, 95% CI 1.28–4.91), knee replacement (OR 1.42, 95% CI 1.00–2.02), blood loss (standardized mean difference (SMD) 0.30, 95% CI 0.15–0.44), dementia (OR 3.09, 95% CI 2.10–4.56), neurologic disorders (OR 2.26, 95% CI 1.23–4.15), psychiatric illness (OR 2.74, 95% CI 1.34–5.62), and obstructive sleep apnea (OR 4.17, 95% CI 1.72–10.09) along with several comorbidity evaluation scores and laboratory markers.

**Conclusion:**

We identified risk factors consistently associated with the incidence of POD in knee and hip replacement. Strategies and interventions should be implemented to the patients receiving knee or hip replacement with potential risk factors identified in this meta-analysis.

**Supplementary Information:**

The online version contains supplementary material available at 10.1186/s13018-020-02127-1.

## Background

Knee and hip replacement surgeries have been performed for decades and are the most common procedures in the orthopedic department. Annually, there would be 512,000 hip replacement and 700,000 knee replacement in the USA [1, 2], and the demand continues to grow worldwide for the outcomes are satisfying based on pain relief and function improvement. Mainly, these procedures are performed in the age group of those older than 60 years [1, 2]. However, complications may disturb the rehabilitation and outcomes of the procedures, and postoperative delirium (POD) serves as a very common one.

As an acute decline in cognitive function, POD is serious and costly which mainly affects elderly people aged 65 years and older with an incidence rate of 12–51% in orthopedic surgery [3]. It is associated with an increase in mortality and morbidity, prolonged length of hospital stay, and worse surgical outcomes [3, 4]. However, POD is preventable with multi-component and targeted interventions which aim to optimize the mobility, nutrition, orientation, cognitive function, and sleep [5, 6]. Therefore, identifying the patients with the potential risks of developing POD, for whom to receive the specific interventions, is important. Numerous risk factors have been identified in the medical, surgical, and intensive care clinical population, while, to be specific, the risk factors in the knee and hip replacement of POD are still under investigation [3, 7]. In joint replacement surgery, a meta-analysis revealed that 17% of patients who underwent total knee or hip replacement surgery developed POD [8], and a systematic review summarized that general anesthesia, advanced age, history of psychiatric illness, decreased functional status, and specific anesthetic agents would raise the POD rate [9]. However, the existing meta-analysis and systematic reviews have not provided the pooled estimation of risk factors or only summarized the incidence of POD in the knee and hip replacement.

As the elderly population is in the majority of patients undergoing knee or hip replacement which is also at a higher risk of POD, understanding the delirium risk factors may help the surgeons, patients, therapists, and caregivers in providing targeting interventions. The current meta-analysis aims to pool the prevalence and risk factors of POD in patients undergoing knee or hip replacement surgery from existing literature as no formal systematic review or meta-analysis has been performed to date. The result would be helpful and could be used by the clinical team of the joint replacement department in optimizing the perioperative program aiming at lowering the incidence of POD among patients undergoing knee and hip replacement.

## Methods

### Literature search

The PubMed, Ovid MEDLINE, and Ovid EMBASE were used to identify original research published all through March 2020 with following keywords: “joint replacement,” “joint arthroplasty,” “knee replacement,” “knee arthroplasty,” “hip replacement,” “hip arthroplasty,” “TJR,” “TJA,” “TKR,” “TKA,” “THR,” “THA,” “delirium,” and “confusion.” References in identified articles and systematic reviews were scanned manually for possible inclusion.

### Eligibility criteria

The original studies included in this meta-analysis should meet the criteria as follows: (1) only assessing patients who underwent knee or hip replacement, (2) available prevalence or risk factors of POD or data from POD and non-POD patients, (3) used any validated tools (Diagnostic and Statistical Manual of Mental Disorders (DSM) [10], Confusion Assessment Method (CAM) [11], or Delirium Rating Scale (DRS) [12], etc.) for POD assessment; and (4) cohort, case-control, and cross-section studies. Studies assessing delirium after discharge and subsyndromal delirium or any other kind of cognitive impairment were not included.

### Study selection

**Fig. 1:**
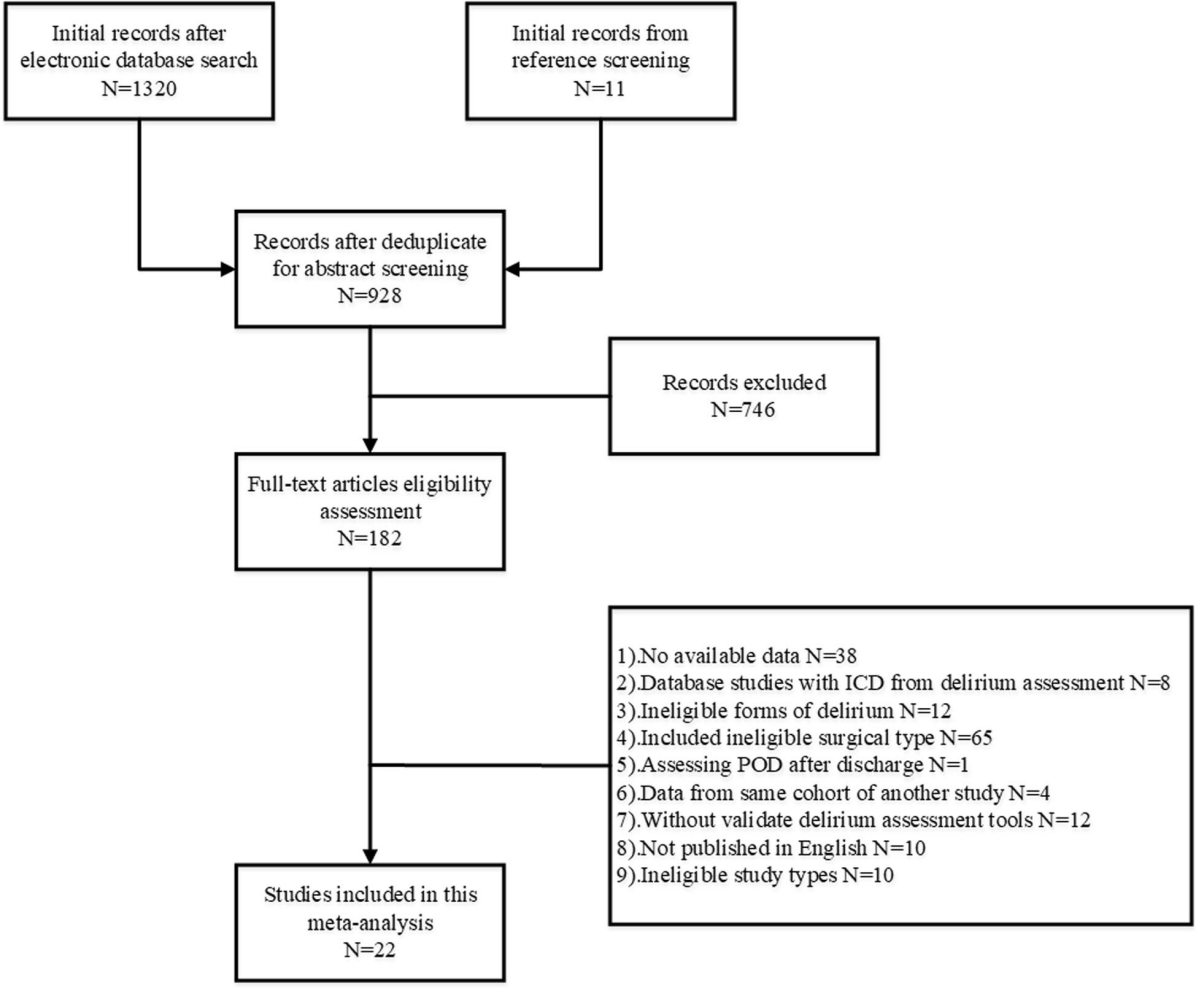
Flow chart of literature search and screening

Endnote was used for two levels of screening by two reviewers. Firstly, we screen the titles and abstracts of the articles based on the inclusion criteria for eligible studies. Secondly, the full-text articles were screened. After that, disagreements of the inclusion and exclusion of the articles were discussed to reach the final agreement between the two reviewers. The flow diagram was presented in Fig. 1 and supplement file 1, and the PRISMA checklist was presented in supplement file 2.

### Data extraction

Two reviewers independently extracted the data from the included studies. At first, the characteristics including titles, authors, year of publication, study design, sample size, type of surgery, and assessment tools of the included studies would be summarized. Then, the risk factors of POD from the included studies were extracted. Importantly, the definitions of each risk factor from different studies were also extracted to prevent misunderstanding. When multiple studies reported the data from the same source, we would adopt the study with the longest follow-up, the largest sample size, or more valid data. Missing data were sought from the corresponding authors where possible. Finally, the disagreements of the extracted data were resolved through discussion.

### Methodological quality assessment

The non-randomized studies included in this meta-analysis were assessed with the Newcastle–Ottawa Scale (NOS) [13] by two independent reviewers. It is a validated and recommended tool for non-randomized studies’ methodological quality describing. The scores of NOS consist of selection criteria, comparability, and outcome (cohort study) or exposure (case-control study). A maximum of 9 scores reveals the highest quality.

### Statistical analysis

The odds ratios (ORs) or standardized mean differences (SMDs) with 95% confidential intervals (CIs) were calculated and pooled where a risk factor was examined by two or more included studies to estimate the association between the risk factors and POD. The between-study heterogeneity was tested with *I*^2^ which was guided by the Cochrane Handbook for Systematic Reviews of Interventions. The random-effect model was used to calculate the pooled ORs or SMDs when significant heterogeneity was presented (*I*^2^ > 50%). And the fixed-effect model was used in the absence of statistical heterogeneity. Incidences of delirium were pooled by the random-effect model which drives an overall effect estimate with 95% CIs. A statistically significant risk factor for POD was considered at a two-tailed *p* value < 0.05. Forest plots were used to summarize the outcomes of meta-analyses. For a risk factor demonstrated by at least 10 studies, we assessed for the potential publication bias with visual funnel plots for symmetry and Eggar’s test. When a statistically significant bias was demonstrated (*p* < 0.05), a trim-and-fill method was used to reveal the missing studies and, adjusting for publication bias, provide a combined effect estimate. All statistical analyses were conducted in StataSE 15.0 using *metan* and associating modules.

## Result

### Characteristics of studies

One thousand three hundred twenty abstracts and titles were included from the initial electronic search. After deduplication, 928 abstracts were scanned based on eligibility criteria which excluded 746 studies and the remaining 182 were screened with full text (Fig. 1). Finally, 22 studies (Table 1) [14–35] with a total of 11934 patients were included in this meta-analysis and in which 1841 cases were found with POD. Four studies [36–39] were excluded due to sharing the same cohorts with included ones [15, 17, 27] which derived the longest follow-up. The result of methodological assessment demonstrated a moderate to high quality of the included studies (Table 2) in which four studies scored 9 [15, 24, 28, 29], seven studies scored 8 [14, 18, 19, 22, 23, 25, 27], five studies scored 7 [17, 26, 30–32], four studies scored 6 [16, 20, 21, 34], and two studies scored 5 [33, 35]. A total of 28 potential risk factors were initially summarized in this meta-analysis (Table 3).

**Table 1:** Characteristics of the included studies

Author	Year	Study design	Type of surgery	Age	Sample size	Case of delirium	Incidence	Gender female/male	Criteria for delirium	Study quality (stars)
de Jong	2019	Case control	Hip	81 ± 8	463	121	26.1%	310/206	DSM-IV	6
Guo^a^	2019	Prospective cohort	Hip	65–80	244	60	24.6%	67/53^a^	CAM	9
Peng	2019	Prospective cohort	Knee and hip	65–85	272	55	20.2%	157/115	DSM-V	8
Cunningham	2019	Prospective cohort	Knee and hip	≥ 65	282	40	14.2%	159/123	CAM	7
Tak kyu	2018	Case control	Knee	71.0 ± 6.9	6020	992	16.5%	5511/509	CAM-ICU	8
Choi	2017	Prospective cohort	Hip	≥ 70	356	110	30.9%	290/66	CAM and CAM-ICU	8
Xin	2017	Prospective cohort	Hip	> 65	120	30	25.0%	58/62	Nu-DESC	8
Wang	2017	Case control	Knee	> 65	265	49	18.5%	244/21	CAM	6
Culley	2017	Prospective cohort	Knee and hip	≥ 65	211	14	6.6%	127/84	CAM	8
Huang	2017	Case control	Knee	N/A^b^	1016	6	0.6%	827/189	DSM-IV	6
Yen	2016	Prospective cohort	Knee	≥ 65	98	22	22.4%	51/47	CAM and DRS-R98	9
Guo	2016	Prospective cohort	Hip	≥ 65	572	120	21.0%	366/206	CAM	8
Chung	2015	Case control	Knee	> 65	365	11	3.0%	332/33	CAM and DSM-IV	7
Cerejeira	2013	Prospective cohort	Hip	≥ 60	101	37	36.6%	51/50	CAM and DSM-IV-TR	8
Flink	2012	Prospective cohort	Knee	≥ 65	106	27	25.5%	59/47	CAM, DSM-IV, and DRS-R-98	9
Jankowski	2011	Prospective cohort	Knee and hip	≥ 65	418	42	10.0%	212/206	CAM	9
Lowery	2008	Prospective cohort	Knee and hip	> 70	94	14	14.9%	53/41	CAM	7
Priner	2008	Prospective cohort	Knee and hip	73.6 ± 6.6	101	15	14.9%	58/43	CAM	7
Wacker^a^	2006	Case control	Knee and hip	≥ 60	572	31	5.4%	20/9^a^	DSM-IV	7
Freter	2005	Prospective cohort	Knee and hip	> 65	132	18	13.6%	88/44	CAM	5
Fisher	1995	Prospective cohort	Knee and hip	≥ 60	80	14	17.5%	43/37	CAM	6
Rogers	1989	Prospective cohort	Knee and hip	≥ 60	46	13	28.3%	31/15	DSM-III	5

*Abbreviations*: *DSM* Diagnostic and Statistical Manual of Mental Disorders, *CAM* Confusion Assessment Method, *ICU* intensive care unit, *Nu-DESC* Nursing Delirium Screening Scale, *DRS-R98* Delirium Rating Scale-Revised-98

^a^Data other than incidence were presented in baseline matched cohorts

^b^Information of age was separately reported for POD and non-POD groups

**Table 2:** The quality of included studies, assessed by Newcastle–Ottawa Scale

Author, year	Study type	Selection				Comparability	Outcome			Overall
		Representative exposed cohort	Selection of the non-exposed cohort	Ascertainment of exposure	Outcome not present at start	Comparability based on design or analysis	Assessment of outcome	Follow-up long enough for outcomes to occur	Adequacy of follow-up of cohorts	
Peng 2019 [14]	Cohort study	*	*	*	*	**	*	*	*	8
Guo 2019 [15]	Cohort study	*	*	*	*	**	*	*	*	9
Cunningham 2019 [17]	Cohort study	*	*	*		**	*	*		7
Xin 2017 [19]	Cohort study	*	*	*	*	**	*	*		8
Culley 2017 [22]	Cohort study	*	*	*		**	*	*	*	8
Yen 2016 [24]	Cohort study	*	*	*	*	**	*	*	*	9
Guo 2016 [25]	Cohort study	*	*	*		**	*	*	*	8
Cerejeira 2013 [27]	Cohort study	*	*	*	*	**	*	*		8
Flink 2012 [28]	Cohort study	*	*	*	*	**	*	*	*	9
Jankowski 2011 [29]	Cohort study	*	*	*	*	**	*	*	*	9
Priner 2008 [30]	Cohort study	*	*	*		**	*	*		7
Lowery 2008 [31]	Cohort study	*	*	*		*	*	*	*	7
Freter 2005 [33]	Cohort study	*	*	*		*	*			5
Fisher 1995 [34]	Cohort study	*	*	*		*	*	*		6
Rogers 1989 [35]	Cohort study	*	*	*			*	*		5
Author year	Study type	Selection				Comparability	Exposure			Overall
		Adequate case definition	Representativeness of the cases	Selection of the controls	Definition of the controls	Comparability based on design or analysis	Ascertainment of exposure (blinding)	Same method of ascertainment for subjects	Non-response rate	
de Jong 2019 [16]	Case-control study	*	*	*	*		*	*		6
Tak kyu 2018 [18]	Case-control study	*	*	*	*	*	*	*	*	8
Wang 2017 [20]	Case-control study	*	*	*	*	*			*	6
Huang 2017 [21]	Case-control study	*	*	*	*		*	*		6
Choi 2017 [23]	Case-control study	*	*	*	*	*	*	*	*	8
Chung 2015 [26]	Case-control study	*	*	*	*	*	*	*		7
Wacker 2006 [32]	Case-control study	*	*	*	*	**		*		7

**Table 3:** Information of all the potential risk factors for delirium after knee and hip replacement

Potential risk factors	No. of studies	Pooled ORs or SMDs	95% CI	*p* value	*I*^2^
Age	15	0.43^a^	(0.24, 0.61)	< 0.001	73.4%
	11	1.15	(1.08, 1.22)	< 0.001	63.5%
Female (compared with male)	14	0.91	(0.82, 1.18)	0.851	49.0%
BMI	7	− 0.22^a^	(− 0.55, 0.12)	0.207	81.4%
Education (years)	6	− 0.18^a^	(− 0.35, − 0.02)	0.026	0.0%
MMSE	11	− 0.32^a^	(− 0.52, − 0.13)	0.001	57.1%
Cognitive impairment	3	6.84	(3.27, 14.33)	< 0.001	0.0%
Dementia	5	3.09	(2.10, 4.56)	< 0.001	38.6%
Neurological disorders	5	2.26	(1.23, 4.15)	< 0.001	0.0%
Psychiatric illness	2	2.74	(1.34, 5.62)	0.006	0.0%
Obstructive sleep apnea	2	4.17	(1.72, 10.09)	0.002	0.0%
Knee replacement	6	1.42	(1.00, 2.02)	0.048	9.8%
Duration of surgery	7	0.29^a^	(0.06, 0.52)	0.013	57.4%
Blood loss	4	0.30^a^	(0.15, 0.44)	< 0.001	0.0%
Spinal anesthesia (compared with general)	5	0.62	(0.46, 0.85)	0.003	45.7%
History of cerebrovascular events	4	2.51	(1.28, 4.91)	0.007	58.4%
ASA ≥ 3	8	1.59	(1.25, 2.03)	< 0.001	29.3%
CCI	5	0.35^a^	(0.04, 0.66)	0.029	56.9%
Length of stay	6	0.53^a^	(0.20, 0.87)	0.002	56.7%
Total protein	2	− 0.68^a^	(− 0.87, − 0.48)	< 0.001	0.0%
Albumin	2	− 0.77^a^	(− 1.36, − 0.19)	0.009	71.6%
Hemoglobin	6	− 0.58^a^	(− 1.11, − 0.04)	0.034	94.4%
Creatine	3	0.16^a^	(− 0.00, 0.32)	0.051	0.0%
C-Reactive protein	2	0.76^a^	(− 0.88, 2.41)	0.363	96.2%
Blood glucose	2	0.41^a^	(− 0.67, 1.51)	0.463	91.7%
Alcohol abuse	3	0.69	(0.32, 1.46)	0.329	76.7%
Diabetic mellitus	8	1.09	(0.86, 1.40)	0.476	29.7%
Hypertension	7	1.06	(0.88, 1.29)	0.522	0.0%
Pulmonary disorders	3	1.13	(0.70, 1.82)	0.61	0.0%
Tobacco usage	3	1.22	(0.68, 2.19)	0.507	0.0%

*Abbreviations*: *OR* odds ratio, *SMD* standardized mean difference, *CI* confident interval, *BMI* body mass index, *MMSE* Mini-Mental State Examination, *ASA* American Society of Anesthesiologists, *CCI* Charlson Comorbidity Index

^a^Data represents pooled SMD

### Incidence of POD

**Fig. 2:**
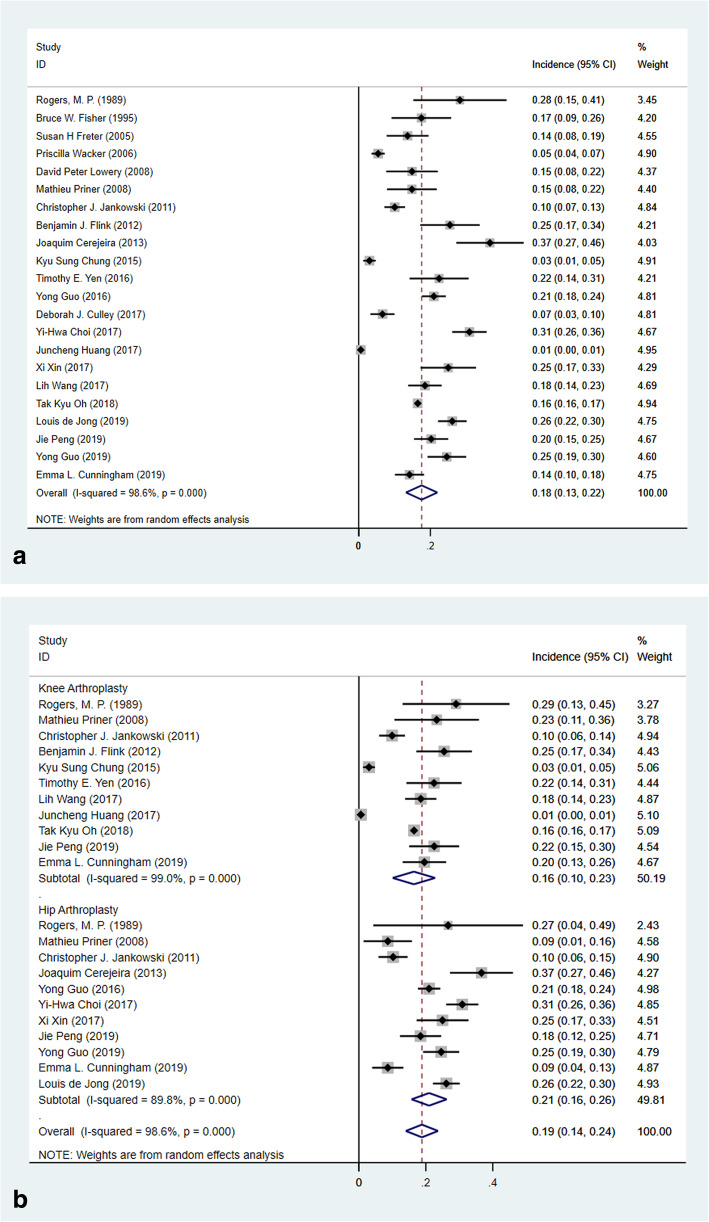
Forest plots of the meta-analysis of the incidence of POD. **a** Incidence of POD of overall patients. **b** Incidence of POD of knee or hip replacement groups

All the included studies reported the incidence of POD which range from 0.6 to 36.6%. A pooled incidence of POD was 17.6% (Fig. 2a, 22 studies, 11934 patients, 95% CI 13.2–22.0%, *I*^2^ = 98.6%). Separately, for knee replacement, a pooled incidence was 16.4% (Fig. 2b, 11 studies, 8439 patients, 95% CI 10.1–22.8%, *I*^2^ = 99.0%), and for hip replacement, it was 18.8% (Fig. 2b, 11 studies, 2406 patients, 95% CI 15.8–26.2%, *I*^2^ = 89.8%).

### Risk factors

#### Advanced age

**Fig. 3:**
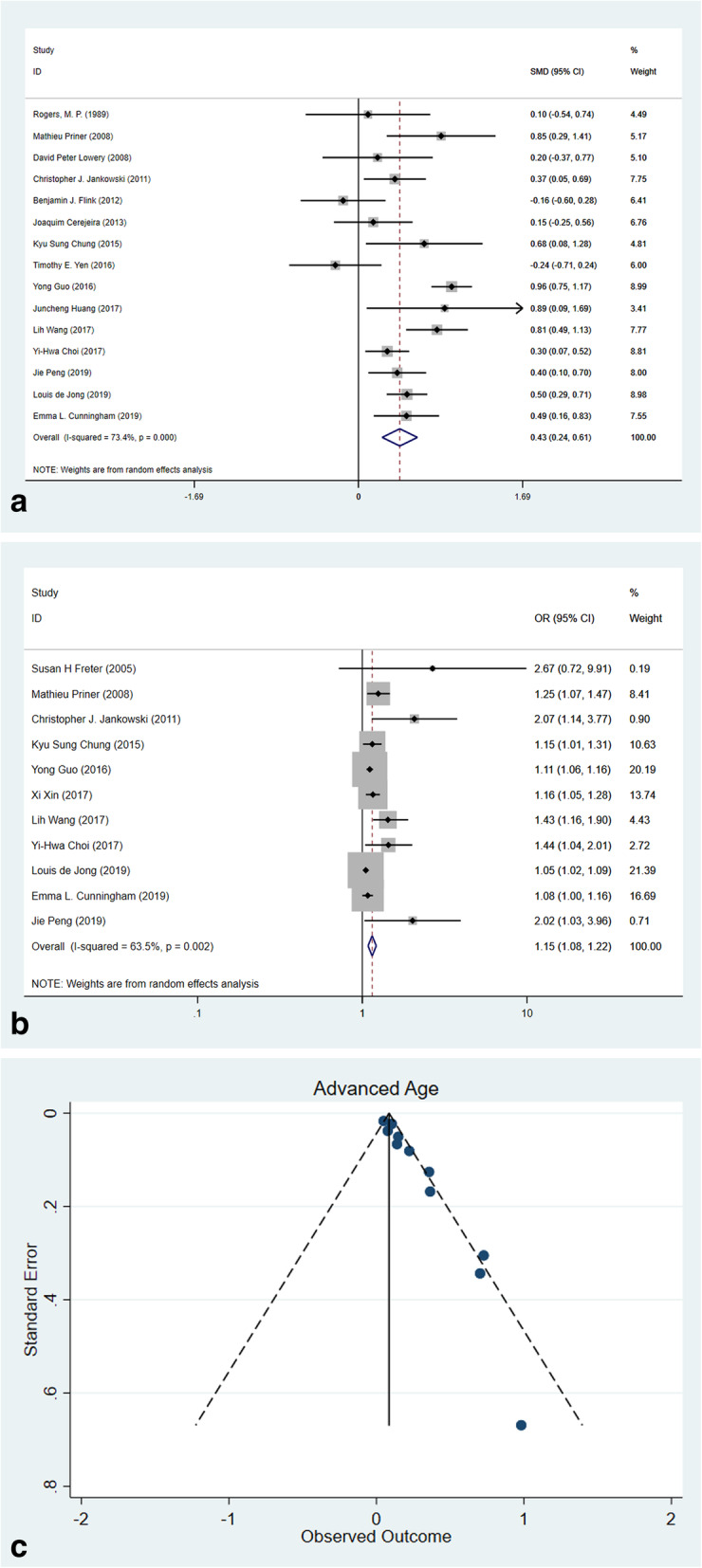
Forest plots and funnel plot of meta-analysis of advanced age. **a** Forest plot of SMD of age. **b** Forest plot of OR of advanced age. **c** Funnel plot of published studies reporting advanced age as a prognostic factor for POD (Egger’s test *p* < 0.001)

A total of 15 studies reported the mean age in POD and non-POD groups, and a pooled SMD revealed that POD patients were mildly older (Fig. 3a, SMD 0.43 years, 95% CI 0.24–0.61, *p* < 0.001, *I*^2^ = 73.4%) than the non-POD patients. Further, 11 studies reported advanced age as a prognostic factor for POD with a pooled OR of 1.15 (Fig. 3b, 95% CI 1.08–1.22, *p* < 0.001, *I*^2^ = 63.5%). Funnel plots showed a significant asymmetry (Fig. 3c, Eggar’s *p* < 0.001). Trim-and-fill revealed that after adjusting for publication bias, the pooled OR remains at 1.12 (95% CI 1.05–1.19, *I*^2^ = 64.4% *p* < 0.001, 5 filled) which sustained the effect estimate was reliable.

#### Cognitive impairment

**Fig. 4:**
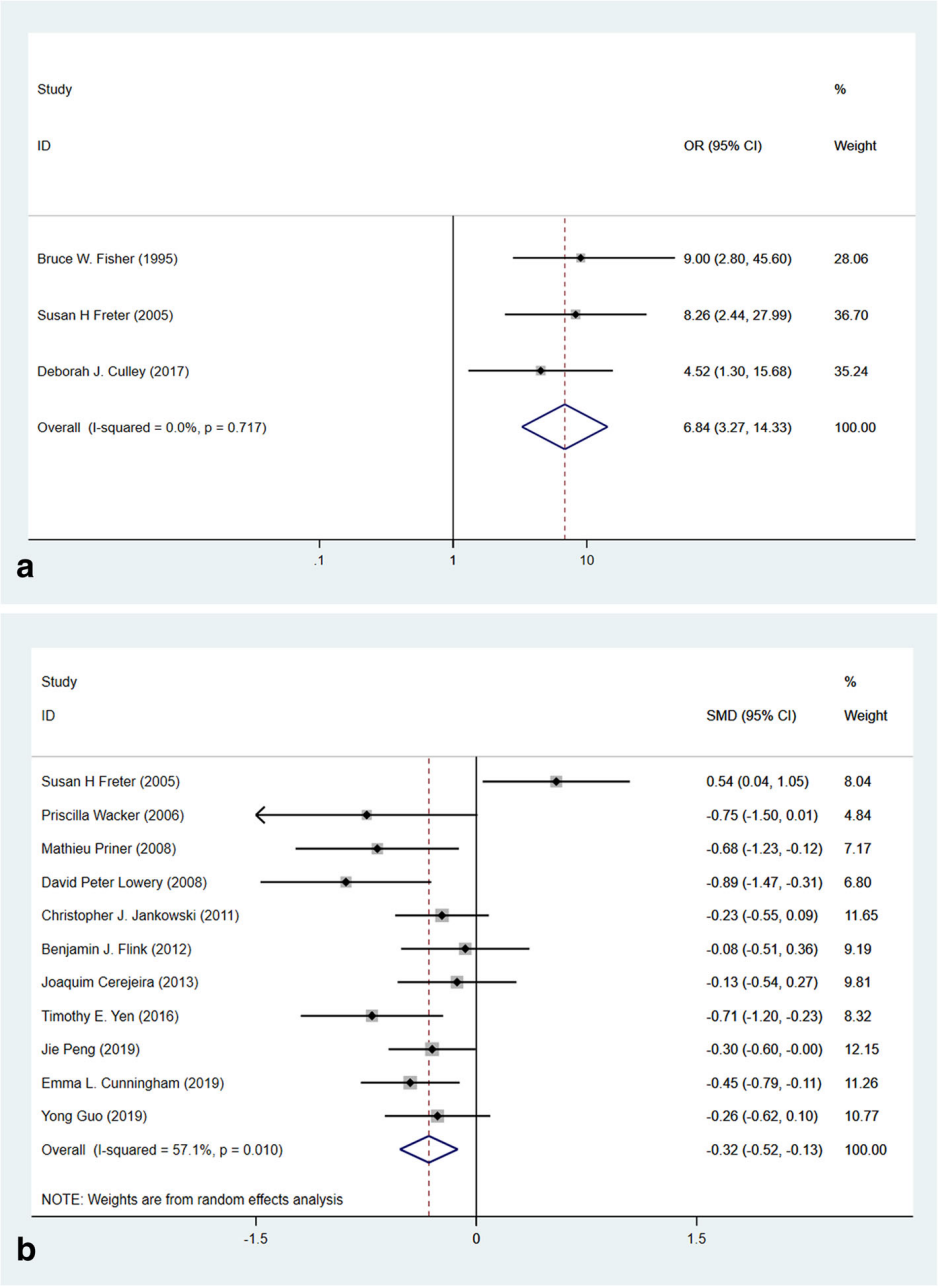
Forest plots of risk factors of cognitive impairment and preoperative MMSE evaluations. **a** Forest plot of cognitive impairment. **b** Forest plot of preoperative MMSE evaluation

As a recognized risk factor for POD, most studies excluded patients with cognitive impairment for removing confounding factors. Three studies reported the patients with cognitive impairment were at higher risk of POD (Fig. 4a, OR 6.84, 95% CI 3.27–14.33, *p* < 0.001, *I*^2^ = 0.0%) without heterogeneity. The Mini-Mental State Examination (MMSE) test is a 30-point questionnaire which is used to measure the cognitive impairment [40]; any score less than 24 indicates an abnormal cognition. In this meta-analysis, a pooled estimate from eleven studies revealed that the MMSE score in the POD group was significantly lower (Fig. 4b, SMD–0.32, 95% CI (− 0.52, 0.13), *p* = 0.001, *I*^2^ = 57.1%) without publication bias (Eggar’s *p* = 0.455).

#### History of cerebrovascular events

**Fig. 5:**
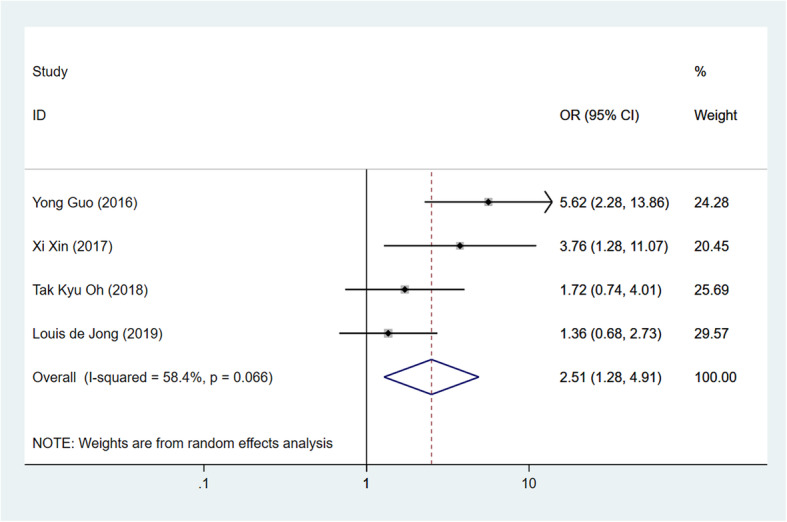
Forest plot of history of cerebrovascular events as a risk factor

Cerebrovascular events, stroke, and transient ischemia, etc., were defined as prognostic factors for POD [3]. Four of the included researches paid attention, and the pooled OR was 2.51 (Fig. 5, 95% CI 1.28–4.91, *I*^2^ = 58.4%, *p* = 0.007) with statistical significance and mild heterogeneity.

#### Medical comorbidities

**Fig. 6:**
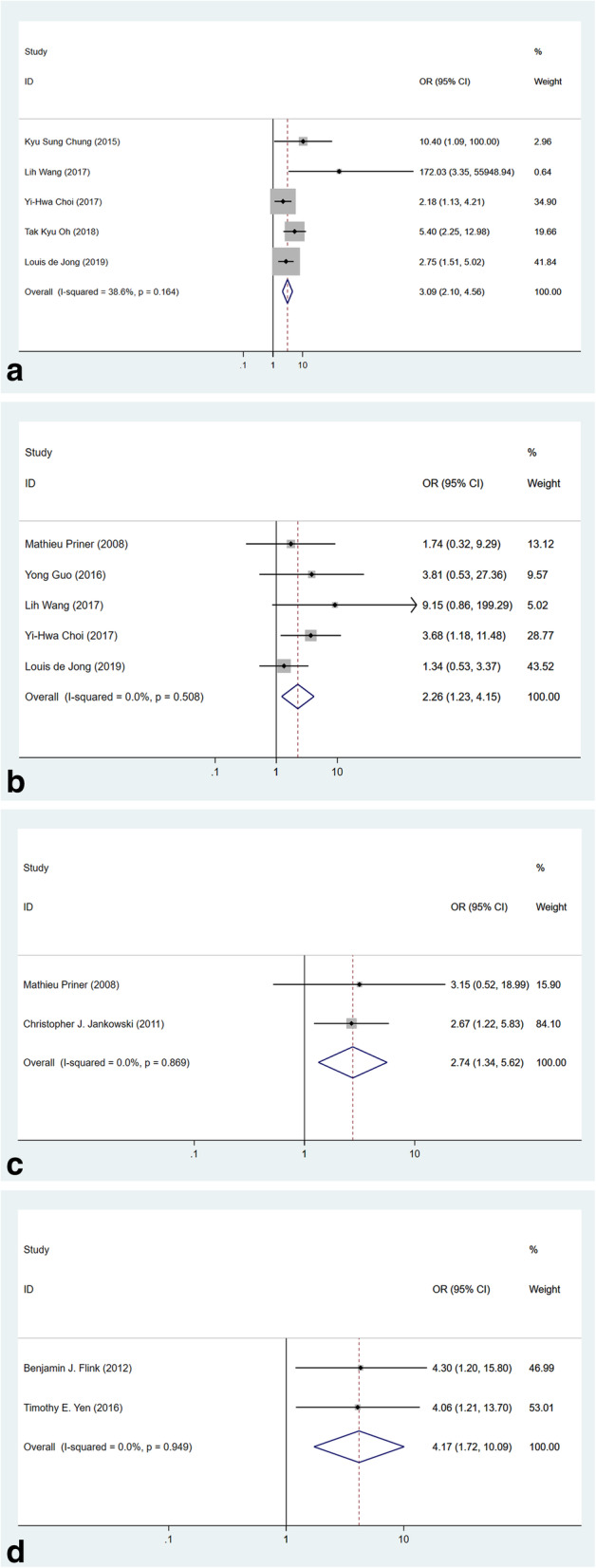
Forest plots of risk factors of medical comorbidities. **a** Forest plot of dementia. **b** Forest plot of neurological disorders. **c** Forest plot of psychiatric illness. **d** Forest plot of obstructive sleep apnea

Common neuropsychiatric comorbidities were reported in certain studies. Dementia was identified as a prognostic factor for POD [3], and our result demonstrated dementia was a significant risk factor for POD with a pooled OR of 3.09 (Fig. 6a, 95% CI 2.10–4.56, *p* < 0.001, *I*^2^ = 38.6%, 5 studies). Further, neurological disorders (Parkinson’s disease, etc.) were reported in five studies and the result of meta-analysis revealed an OR of 2.26 (Fig. 6b, 95% CI 1.23–4.15, *p* < 0.001, *I*^2^ = 0.0%). Last but not the least, patients with psychiatric illness were evaluated in two studies. The pooling of data derived an OR of 2.74 (Fig. 6c, 95% CI 1.34–5.62, *p* = 0.006, *I*^2^ = 0.0%) which confirmed the prognostic effect of psychosis.

Association has been found between sleep disturbance and POD [41]. The pooling of data from two studies which demonstrated the data between obstructive and POD derived an OR of 4.17 (Fig. 6d, 95% CI 1.72–10.09, *p* = 0.002, *I*^2^ = 0.0%) with statistical significance.

#### Surgical and anesthesia factors

**Fig. 7:**
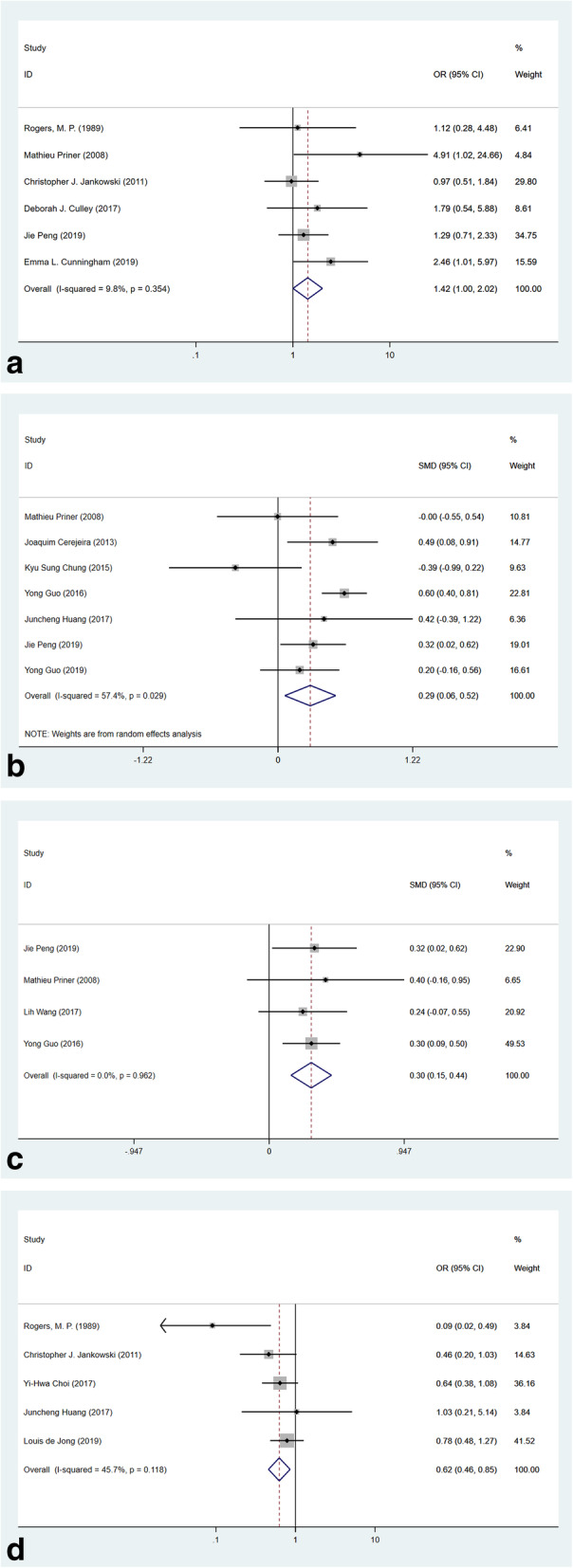
Forest plots of surgical and anesthetic risk factors. **a** Forest plot of knee replacement (compared with hip replacement). **b** Forest plot of duration of surgery. **c** Forest plot of blood loss. **d** Forest plot of spinal anesthesia (compared with general anesthesia)

Performing knee or hip replacement may result in a different rate of POD. Six studies reported the POD incidence in the knee and hip replacement cohort. The meta-analysis result showed that compared with the hip replacement, knee replacement surgery derived an OR of 1.42 (Fig. 7a, 95% CI 1.00–2.02, *p* = 0.048, *I*^2^ = 9.8%) for POD without heterogeneity. Further, the duration of surgery of patients in the POD group was longer (Fig. 7b, SMD 0.29, 95% CI 0.06–0.52, *p* = 0.013, *I*^2^ = 57.4%, 7 studies) and the blood loss was worse (Fig. 7c, SMD 0.30, 95% CI 0.15–0.44, *p* < 0.001, *I*^2^ = 0.0%, 4 studies).

On the other hand, the comparison between anesthesia types was reported by five studies. Patients who underwent spinal anesthesia were less likely to sustain POD when compared with general anesthesia and the combinable OR was 0.62 (Fig. 7d, 95% CI 0.46–0.85, *p* = 0.003, *I*^2^ = 45.7%).

#### Preoperative evaluation of comorbidities

**Fig. 8:**
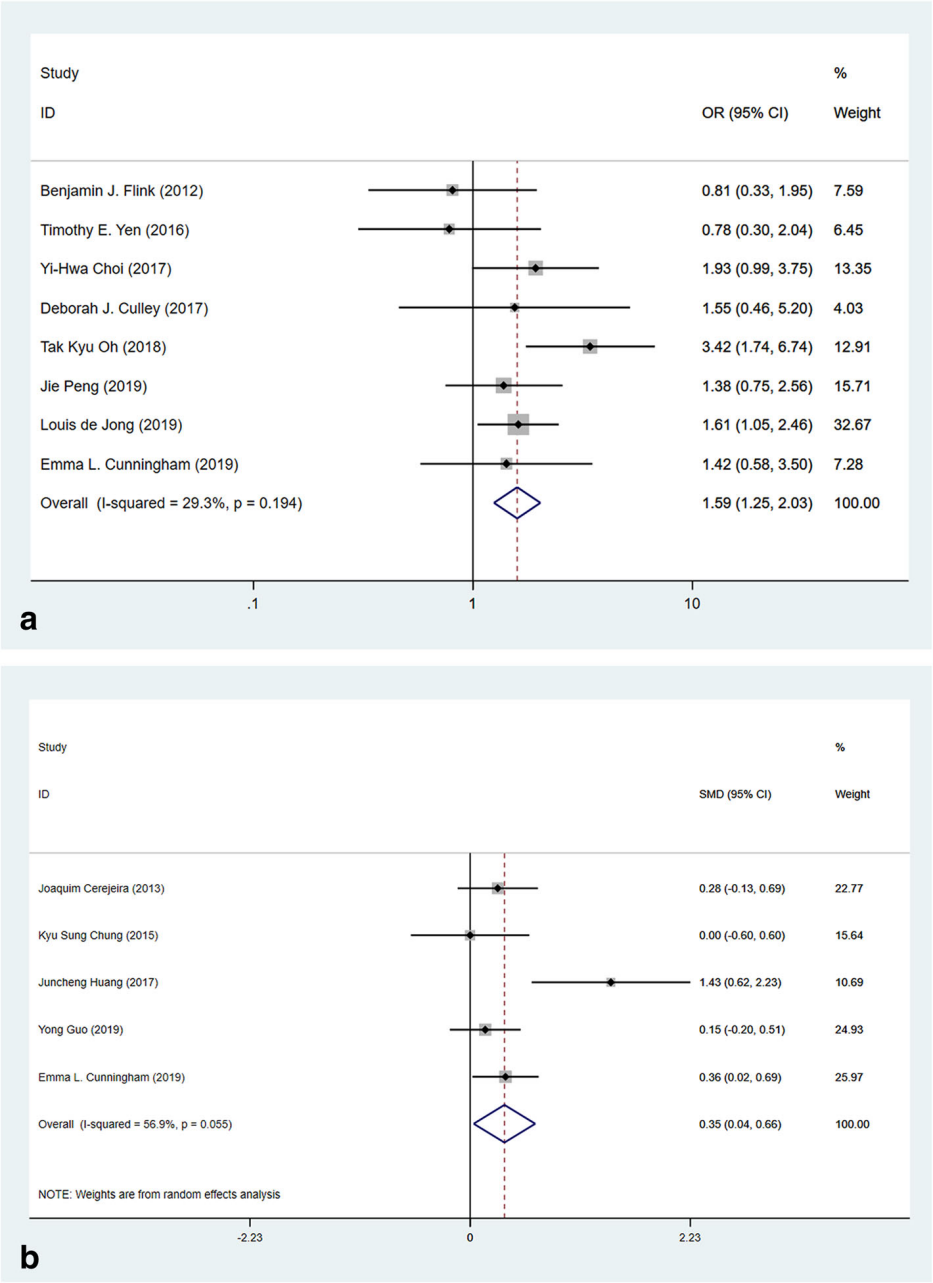
Forest plots of preoperative evaluation of comorbidities in predicting POD. **a** Forest plot of ASA score ≥ III. **b** Forest plot of CCI score

Multiple preoperative evaluations of the patients’ physical status demonstrated differences existing between the POD group and the non-POD group. Eight studies reported that patients with American Society of Anesthesiologists’ (ASA) score III or above were at a higher risk for developing POD (Fig. 8a, OR 1.59, 95% CI 1.25–2.03, *p* < 0.001, *I*^2^ = 29.3%). Further, the Charlson Comorbidity Index (CCI), reported by five studies, was higher in the POD group compared with that in the non-POD group (Fig. 8b, SMD 0.35, 95% CI 0.04–0.66, *p* = 0.029, *I*^2^ = 56.9%).

#### Laboratory test

**Fig. 9:**
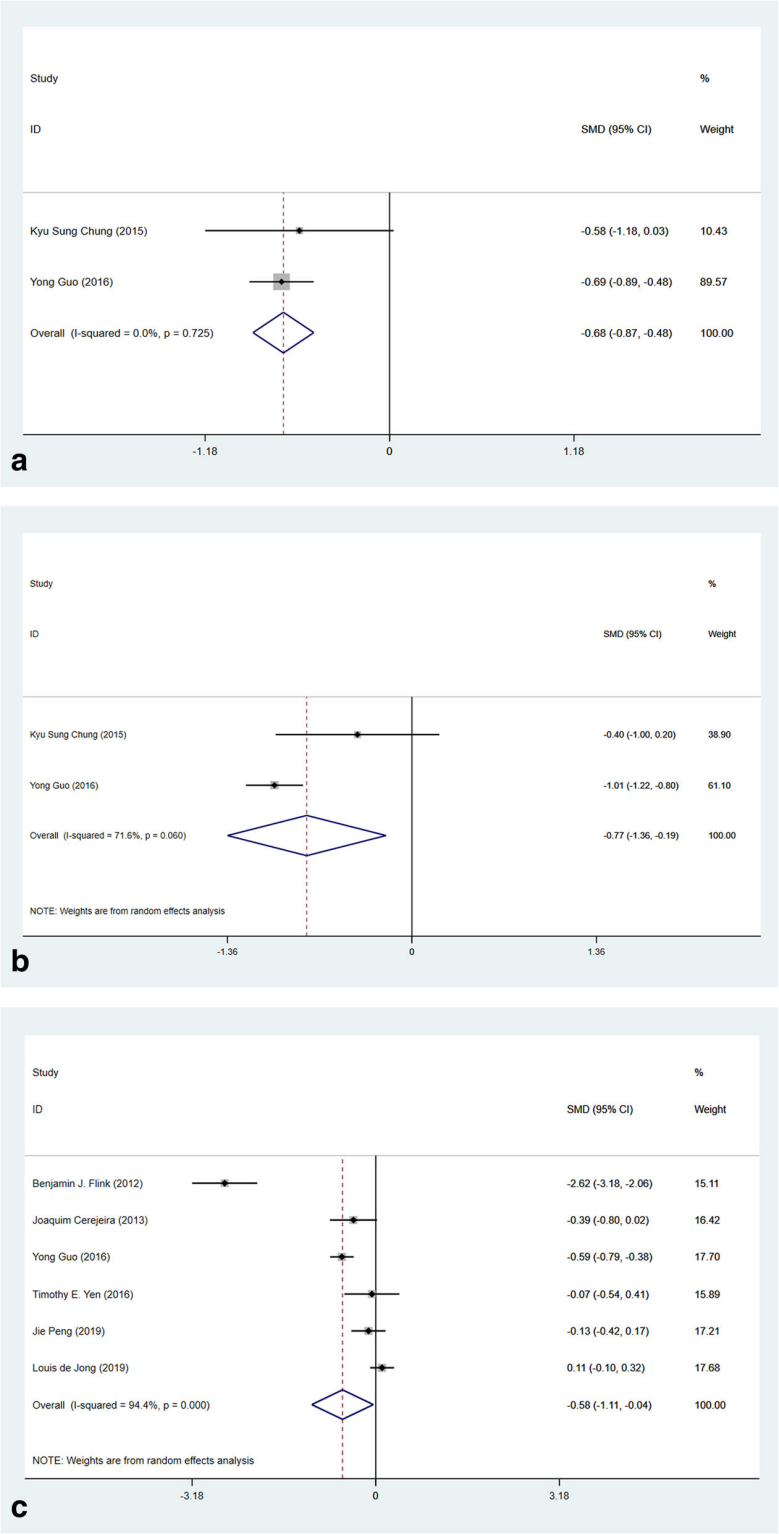
Forest plots of laboratory test in predicting POD. **a** Forest plot of the difference of preoperative total protein. **b** Forest plot of the difference of preoperative albumin. **c** Forest plot of the difference of preoperative hemoglobin

Several preoperative laboratory indicators were presented in certain studies. Patients suffered from POD showed a lower level in total protein (Fig. 9a, 2 studies, SMD − 0.68, 95% CI (− 0.87, − 0.48), *p* < 0.001, *I*^2^ = 0.0%), albumin (Fig. 9b, 2 studies, SMD − 0.77, 95% CI (− 1.36, − 0.19), *p* = 0.009, *I*^2^ = 71.6%), and preoperative hemoglobin (Fig. 9c, 6 studies, SMD − 0.58, 95% CI (− 1.11, − 0.04), *p* = 0.034, *I*^2^ = 94.4%).

## Discussion

The current meta-analysis included 22 studies and presented an incidence of 17.6% (Fig. 2) in knee and hip replacement patients. It was consistent with a previous meta-analysis that concluded 17.3% of patients who underwent total joint replacement would develop POD [8]. We identified 28 potential prognostic factors, and 18 revealed statistically significant. Preoperative factors included advanced age, education, ASA and CCI scores, cognitive impairment, neuropsychiatric comorbidities, obstructive sleep apnea, and history of cerebrovascular events. Surgery-related factors were knee replacement, duration of surgery, blood loss, and general anesthesia. Significance difference existed in some laboratory markers including preoperative total protein, albumin, and hemoglobin. Besides, there was a prolonged length of stay of patients who suffered from POD (SMD − 0.53, 95% CI (0.20, 0.87), *p* = 0.002, *I*^2^ = 56.7%).

Advanced age was a robust prognostic factor. Our study reported that the admission age was 2.7 years older in the POD group with a mild prognostic strength (OR 1.15). The previous researches demonstrated a wide range of OR (1.07–12.95) of advanced age in surgical awards [42–44], and the possible reason was the definition of “advanced age” and surgical types differed between studies. Age-related cerebral changes in stress-regulating neurotransmitter and intracellular signal transduction systems were the main reasons for the development of delirium in elderly patients [45]. Further, ASA ≥ III (OR 1.59) or higher CCI (SMD 0.35) were significant indicators that sustain that patients with a deteriorated physical status were susceptible to POD. A previous meta-analysis also indicated older patients with frailty had more chance of developing POD [46]. Indeed, inflammation and sickness may result in aberrant stress response under the surgical circumstance which is vulnerable to POD [47]. Generally, as the gerontic patients are the main population receiving knee or hip replacement, physical status evaluation should be routinely carried out with much attention paid to the advanced age or functional impaired ones.

Pre-existing cognitive impairment is a chronic, long-term status that may manifest as memory impairment, visuospatial disorders, word-finding difficulties, or changes in attention functions [48] while POD is described as an acute confusional state or altered mental status [49]. Consistent with previous meta-analyses [50, 51], we showed patients with pre-existing cognitive impairment are at a higher risk for developing POD (OR 6.84). The age-related cerebral change with the effects of cerebrovascular events, which was also a prognostic factor proved in the study, can result in susceptibility to delirium when biologically stressed, especially when underlying cognitive impairment exists, not to mention surgical stress [47, 52]. Further, patients with neuropsychiatric disorders were more susceptible to POD due to inflammation, chronic stress, neuronal damage, and impaired cholinergic function [53]. We proved that the MMSE score differs between groups significantly. It highlighted that preoperative cognitive assessment would be effective in screening potential delirium-risk patients which was verified by several studies [22, 30, 33]. Therefore, joint replacement surgeons should put the cognitive assessment in the standardized program for elective surgery [54].

Patients who developed delirium were more often those who underwent knee replacement, with longer surgical time, and suffered worse blood loss. It is unclear why knee replacement is a risk factor of POD. We suppose that firstly, the initial diagnoses of patients who underwent knee replacement were more likely inflammatory disease [1], osteoarthritis and rheumatoid arthritis, etc., and as for hip replacement trauma, dysplasia, or necrosis played a part [2]. And an inflammation state may promote the pathophysiology process of delirium [47]. Secondly, patients may suffer greater pain after knee replacement, and it indicates extension analgesic burdens which may be contributory [55]. Finally, in knee replacement, the utilization of a tourniquet was common but it would increase neuroinflammatory burden via the washout of an ischemic limb [17]. Consistently with a previous study, prolonged duration of surgery may contribute to the development of POD [56]. As theory says the possible pathophysiology of delirium is disruptions of cerebral autoregulation, which can occur during surgery [57], a prolonged surgery would result in exacerbated hypercapnia, hypothermia, and worse blood loss, which all contribute to diminished autoregulation and triggers POD. This result draws attention to the complex knee replacement operation including bilateral replacement and revision. For patients with a potential risk of POD receiving this kind of surgery, thorough preoperative planning by experienced surgeons and anesthetists was essential to guarantee the surgery would be completed safely and quickly.

Spinal anesthesia was associated with reduced OR for POD (OR 0.62). This result is consistent with several previous studies [8, 58, 59]. Recently, two high-volume retrospective population-based studies both revealed a 17–45% decrease of odds when compared with general anesthesia. However, the underlying mechanism remains controversial. The spinal anesthesia has the effect of reducing the delirium risk that may be related to the reduction of systemic anesthetic drugs which affect the central nervous system [60]. A study compared epidural with general anesthesia and found a significant mental change after total hip arthroplasty in the general anesthesia group while no change in the epidural group [61]. The explanation was that general anesthesia may lead to potential hyperventilation, reduced cardiac output, reduced cerebral blood flow, postoperative hypoxemia, and cerebral vasoconstriction which all contribute to the development of POD [61]. This result indicates general anesthesia in knee and hip replacement was a risk factor for POD. Preoperative planning of the anesthetic type to certain patients who are under a potential risk of POD would be indispensable, and, when possible, spinal anesthesia should be chosen.

There were several limitations to this study. First, we used both unadjusted ORs and adjusted ORs to pool the final estimates. Some of the studies reported only unadjusted effect measures, which limited their ability to account for possible confounders. Including these data may result in overestimated results. Second, heterogeneity existed in some outcomes. Due to the limited number of studies, the heterogeneity could not always be adequately explored. However, the random-effect model was used when *I*^2^ > 50%, and the heterogeneity of results summarized from sufficient studies were explored and the reliability was confirmed. Third, the subtypes of the knee or hip replacement surgery were not evaluated in this study. Some included studies reported data from hemiarthroplasty, revision surgeries, or bilateral surgeries. Due to the lack of sufficient original data, we did not perform subgroup analysis.

Our study had several strengths. First, we presented 28 potential risk factors among which 18 were statistically significant while the previous meta-analysis only reported pooled incidence of POD in the knee and hip replacement [8], and the risk factors were summarized in a systematic review without an overall estimation of effects [9], which meant this is by far the first study to quantitatively summarize the risk factors for the postoperative delirium after knee replacement or hip replacement. Second, twenty-two studies and over 11,000 patients were included in this meta-analysis which gave us sufficient data to investigate the possible prognostic factors. Third, every included study was carefully screened with methodology assessment resulting in a moderate to high quality, which meant the extracted data was reliable.

## Conclusion

In summary, this meta-analysis found POD was common in the knee or hip replacement surgery. And we identified 18 significant risk factors from patient-related and operation-related fields. Future efforts should be made to determine the risk factors in each subtype of joint replacement and which risk factors should be paid more attention to and how to quantify them. This meta-analysis suggested joint replacement surgeons perform cognitive assessment preoperatively and look into the reasons for the prognostic strength of knee replacement.

## Supplementary Information

**Additional file 1: Supplement file 1.** PRISMA flow diagram.

**Additional file 2: Supplement file 2.** PRISMA checklist.

## Data Availability

The datasets used and analyzed during the current study are available from the corresponding author on reasonable request.
