# Supplementary material for: Risk factors of postoperative delirium in the knee and hip replacement patients: a systematic review and meta-analysis
Source: J Orthop Surg Res. 2021 Jan 22;16:76. doi: 10.1186/s13018-020-02127-1 (PMC7821501; doi:10.1186/s13018-020-02127-1)
Supplement: Supplementary file 1 — Additional file 1: Supplement file 1. PRISMA flow diagram. [file 13018_2020_2127_MOESM1_ESM.doc]

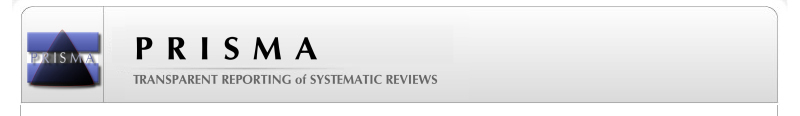
**PRISMA 2009 Flow Diagram**

**Screening**

**Included**

**Eligibility**

**Identification**

Records identified through database searching
(n = 1320)

Additional records identified through other sources
(n = 11)

Records after duplicates removed
(n = 928)

Records screened
(n = 928)

Records excluded
(n = 746)

Full-text articles assessed for eligibility
(n = 182)

Full-text articles excluded, with reasons
(n = 160)

Studies included in qualitative synthesis
(n = 22)

Studies included in quantitative synthesis (meta-analysis)
(n = 22)
